# Supplementary figures and images for: A bacterial ecocline in Klebsiella pneumoniae may explain its backboned phylogeny
Source: PLoS Biol. 2026 Mar 10;24(3):e3003672. doi: 10.1371/journal.pbio.3003672 (PMC12974863; doi:10.1371/journal.pbio.3003672)

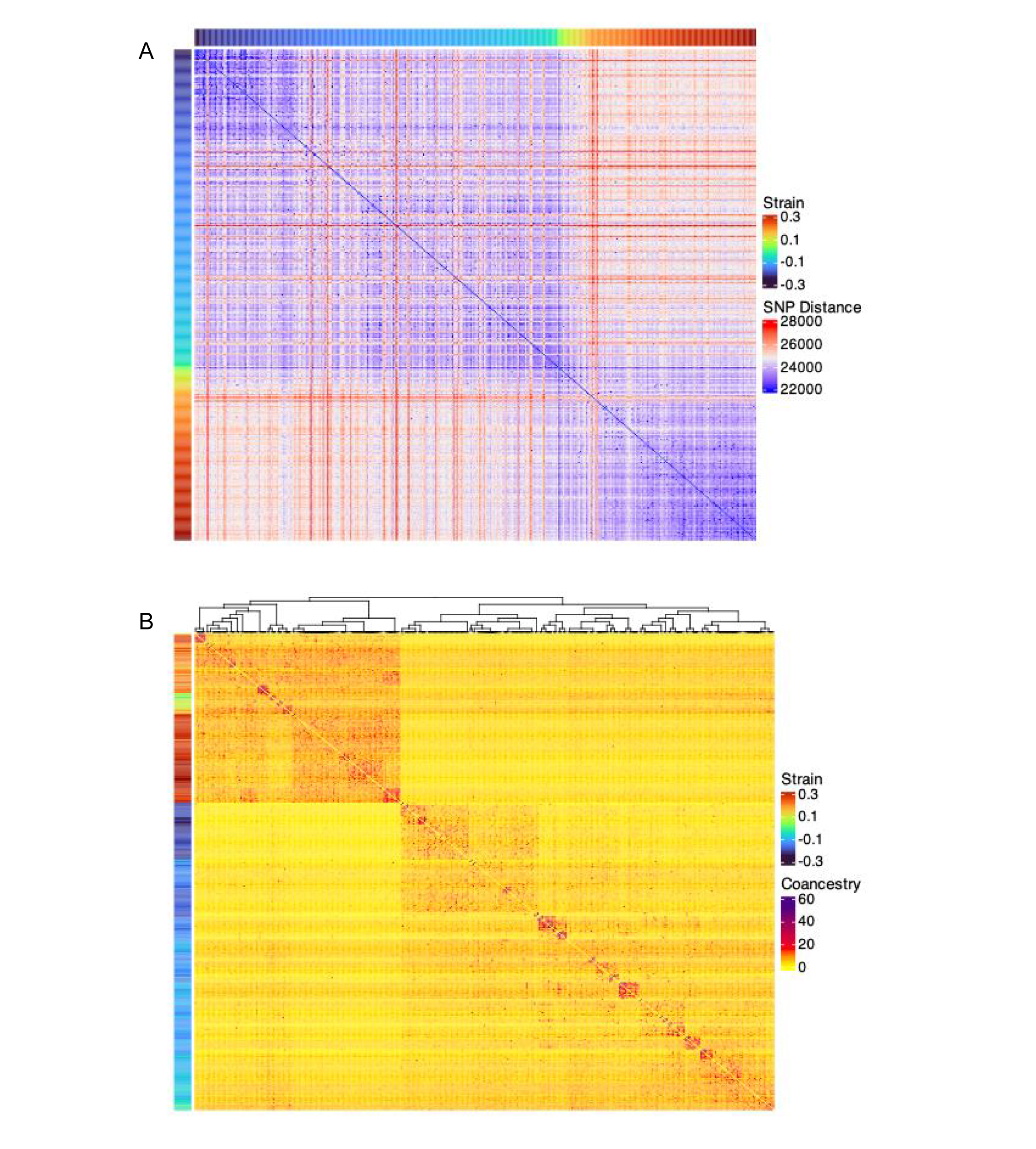

Supplement: S1 Fig — (A) SNP distance matrix illustrating the genetic distances between strains in the non-redundant dataset, with strains ordered based on their PC1 values. The minimum SNP distance observed is 20,000 SNPs. (B) fineSTRUCTURE analysis of the non-redundant KP dataset, highlighting population structure and clustering patterns. The “strain” bar on the left represents the PC1 values of the respective strains. The data underlying this Figure can be found in https://zenodo.org/records/18520201. (TIF) [file pbio.3003672.s005.tif]

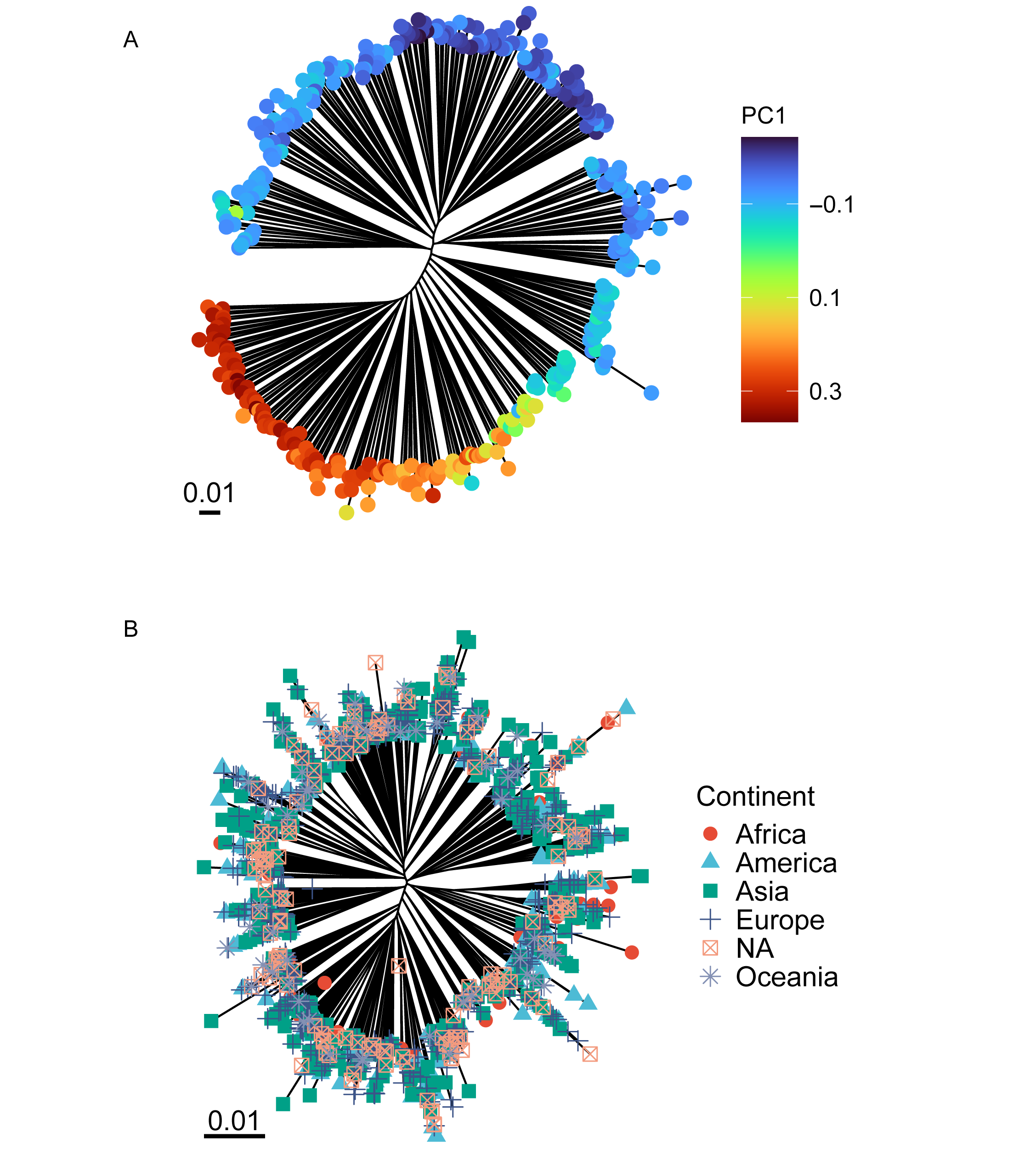

Supplement: S2 Fig — (A) Maximum-likelihood tree of the non-redundant K. pneumoniae dataset generated with IQ-TREE. (B) Maximum-likelihood tree of the complete dataset generated with FastTree. The data underlying this Figure can be found in https://zenodo.org/records/18520201. (TIF) [file pbio.3003672.s006.tif]

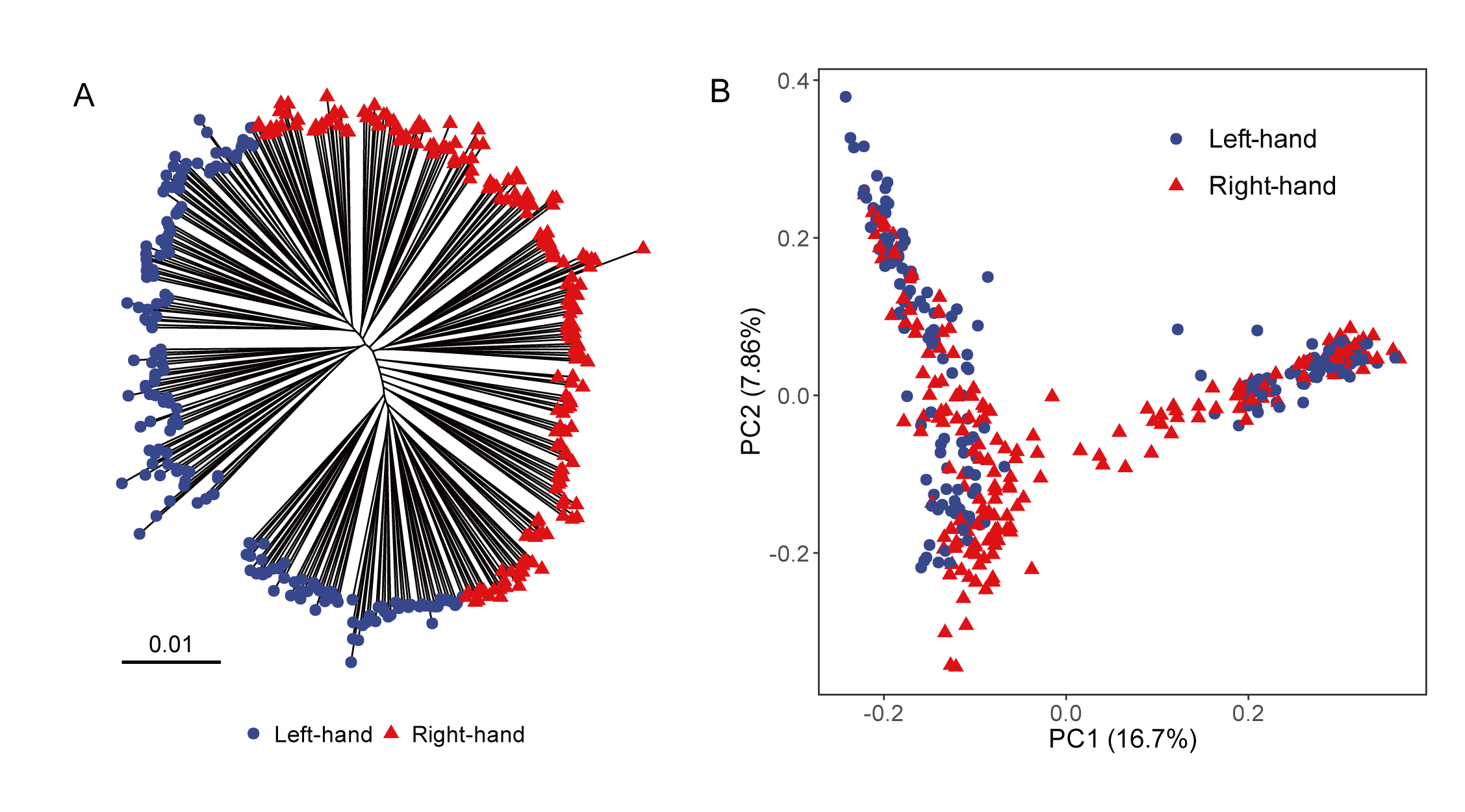

Supplement: S3 Fig — (A) Phylogenetic tree of the non-redundant KP dataset divided into two subsets based on the backbone. (B) PCA of the non-redundant KP dataset colored according to two sub-groups. The data underlying this Figure can be found in https://zenodo.org/records/18520201. (TIF) [file pbio.3003672.s007.tif]

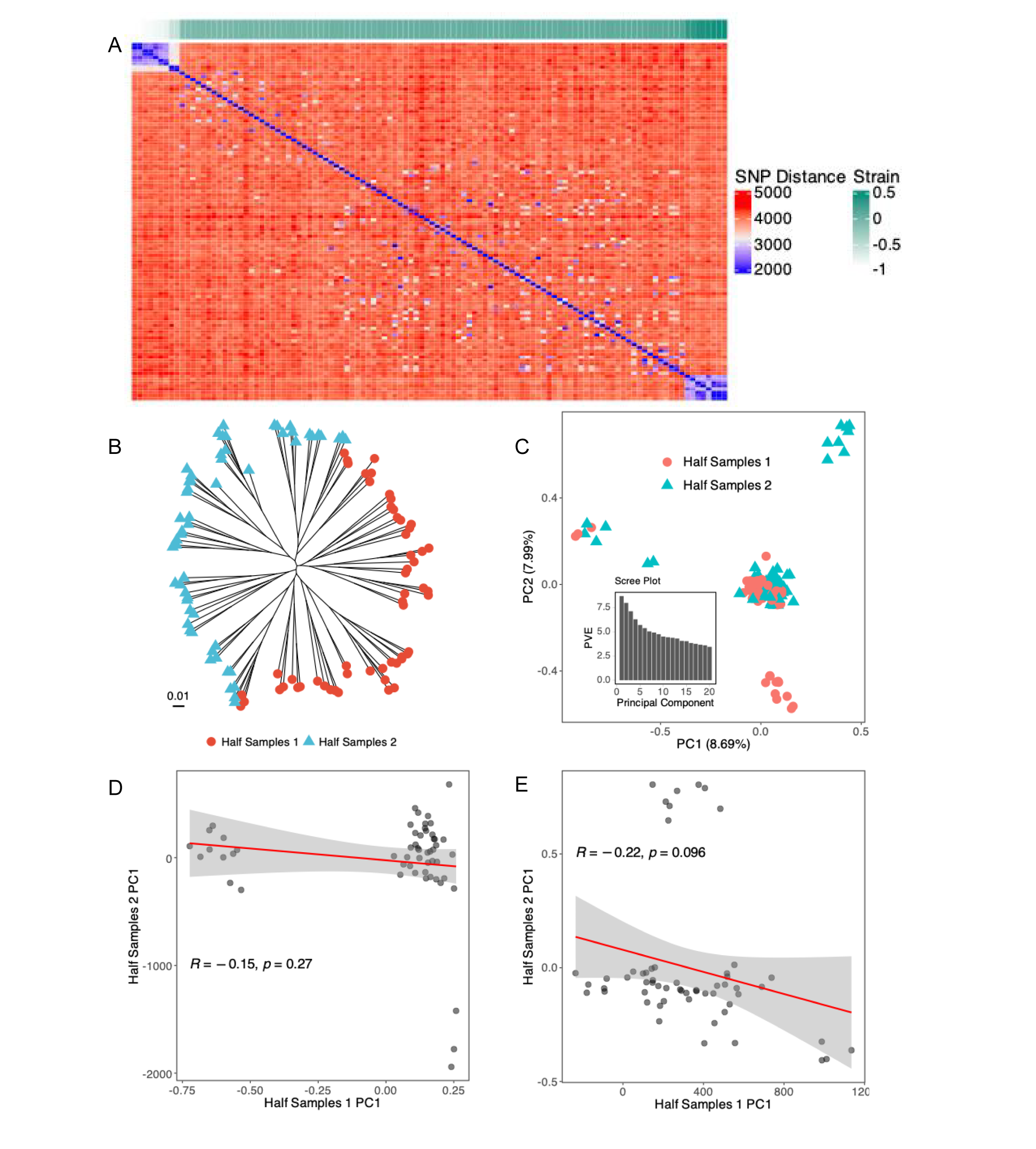

Supplement: S4 Fig — (A) SNP distance matrix for the clonal simulation. The minimum SNP distance observed is 2,000 SNPs. Half-strain matching: (B) Phylogenetic tree of the non-redundant clonal simulation divided into 2 subsets. (C) PCA of the non-redundant clonal simulation data. (D) Correlation of PC1 from group 2 projected onto group 1 results. (E) Correlation of PC1 from group 1 projected onto group 2 results. The data underlying this Figure can be found in https://zenodo.org/records/18520201. (TIF) [file pbio.3003672.s008.tif]

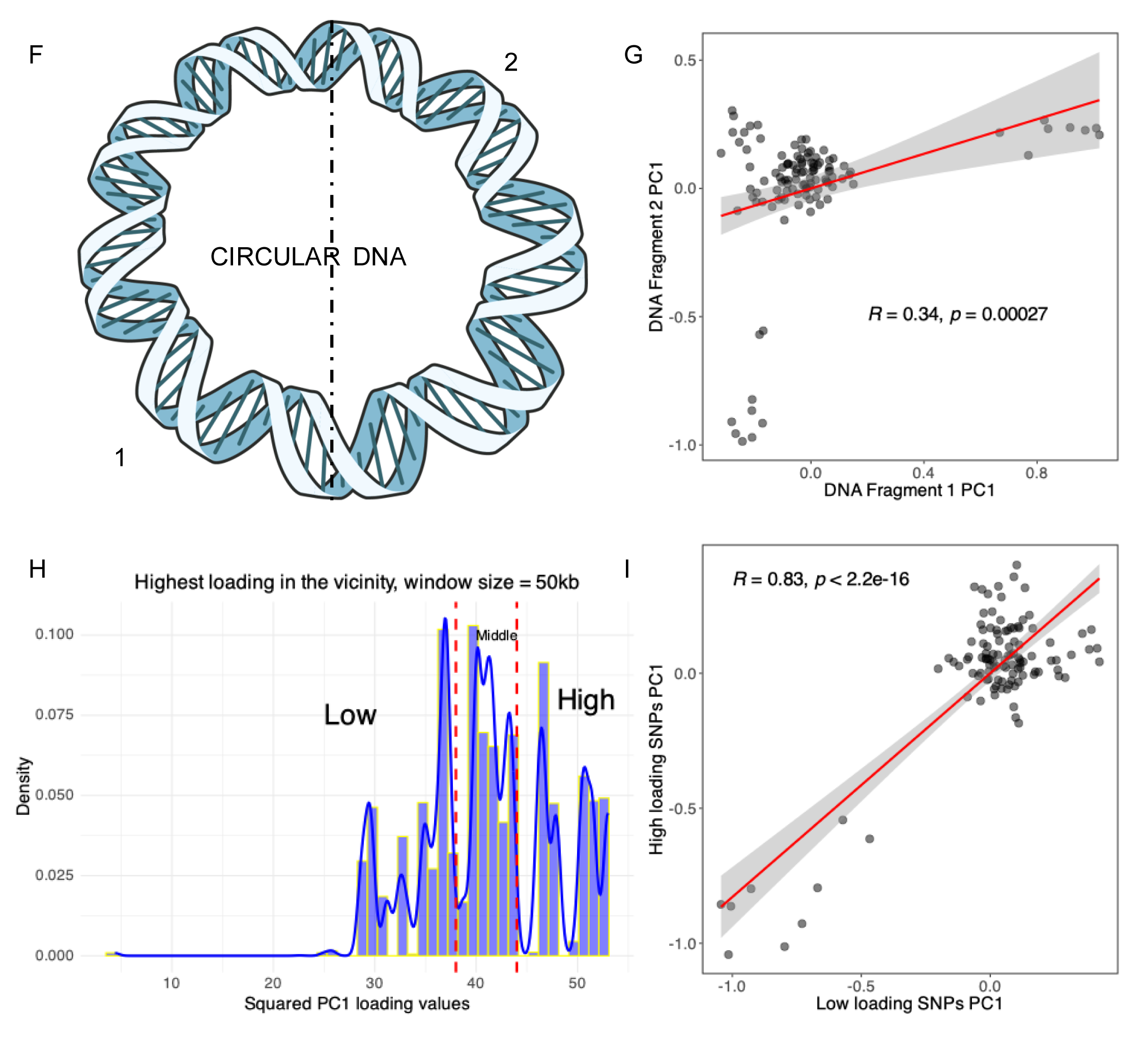

Supplement: S5 Fig — (F) Division of the genome into two fragments (Fragment 1 and Fragment 2). (G) Correlation of PC1 between the two fragments. (H) Histogram of SNP loadings on PC1, dividing SNPs into high- and low-loading groups. (I) Correlation of PC1 between high- and low-loading SNP groups. The data underlying this Figure can be found in https://zenodo.org/records/18520201. (TIF) [file pbio.3003672.s009.tif]

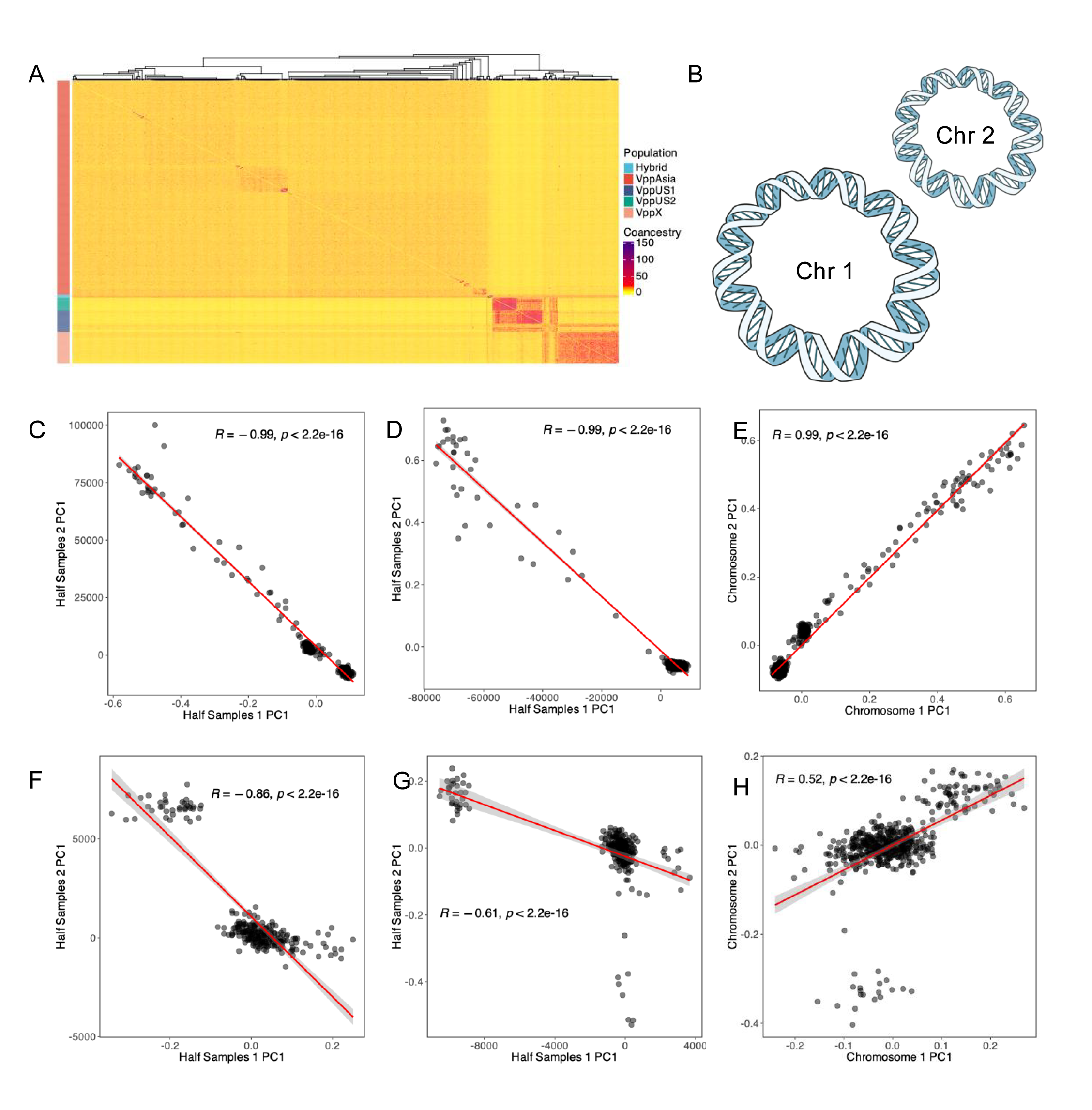

Supplement: S6 Fig — (A) fineSTRUCTURE analysis assigning populations for the non-redundant VP dataset. (B) Representation of VP Chromosome 1 and Chromosome 2, with proportions reflecting their actual sizes. Half-strain matching for the non-redundant VP dataset: (C) Correlation of PC1 from group 2 projected onto group 1 results; (D) Correlation of PC1 from group 1 projected onto group 2 results. Half-chromosome matching for the non-redundant VP dataset: (E) Correlation of PC1 between the two chromosomes. Half-matching for VppAsia dataset: (F) Correlation of PC1 from group 2 projected onto group 1 results; (G) Correlation of PC1 from group 1 projected onto group 2 results. Half-chromosome matching for VppAsia dataset: (H) Correlation of PC1 between the two chromosomes. The data underlying this Figure can be found in https://zenodo.org/records/18520201. (TIF) [file pbio.3003672.s010.tif]

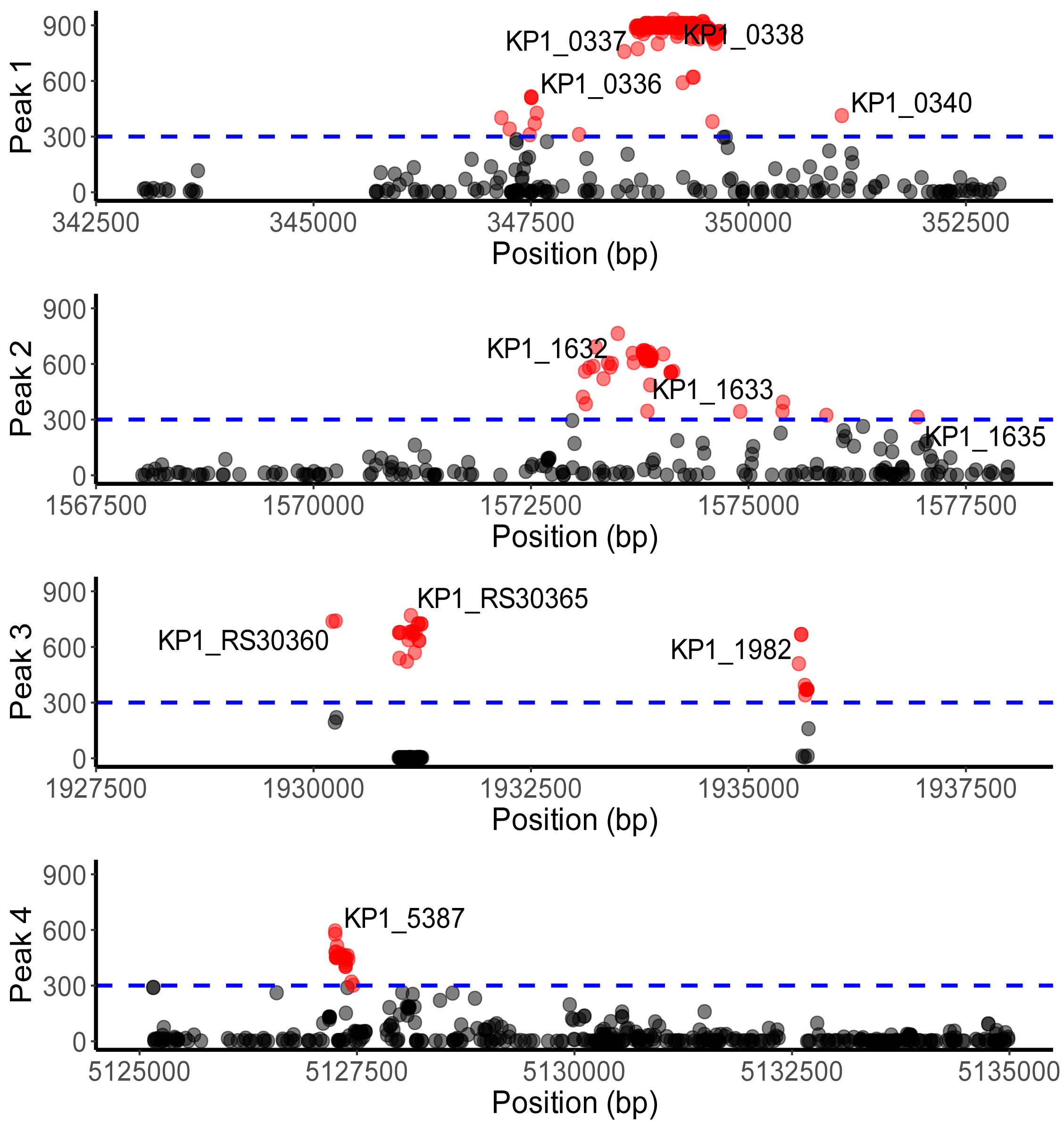

Supplement: S7 Fig — Each panel shows a zoomed-in genomic region corresponding to a high-loading SNP peak, in which multiple SNPs are clustered within a genomic interval of less than 10 kb. The data underlying this Figure can be found in https://zenodo.org/records/18520201. (TIF) [file pbio.3003672.s011.tif]

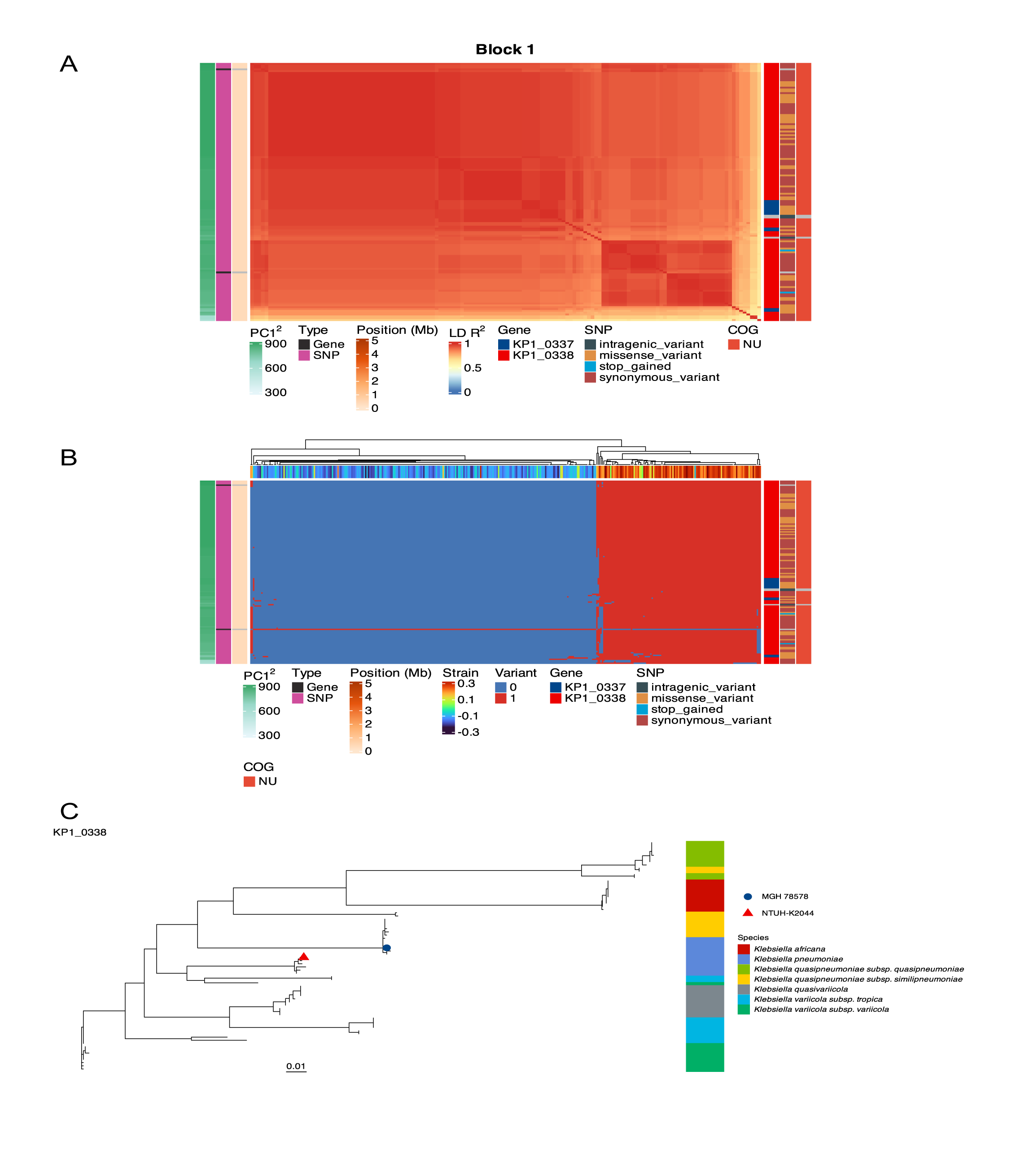

Supplement: S8 Fig — (A) SNPs in KP1_0337, KP1_0338, and associated accessory genes form a high LD block. (B) Variant (1/0) heatmap shows clear differentiation in gene presence/absence or SNP minor/major alleles across isolates. (C) Multi-species phylogenetic tree of the KP1_0338 gene. The annotation strip indicates the species of each strain. COG one-letter code descriptions: [N] Cell motility; [U] Intracellular trafficking, secretion, and vesicular transport. The data underlying this Figure can be found in https://zenodo.org/records/18520201. (TIF) [file pbio.3003672.s012.tif]

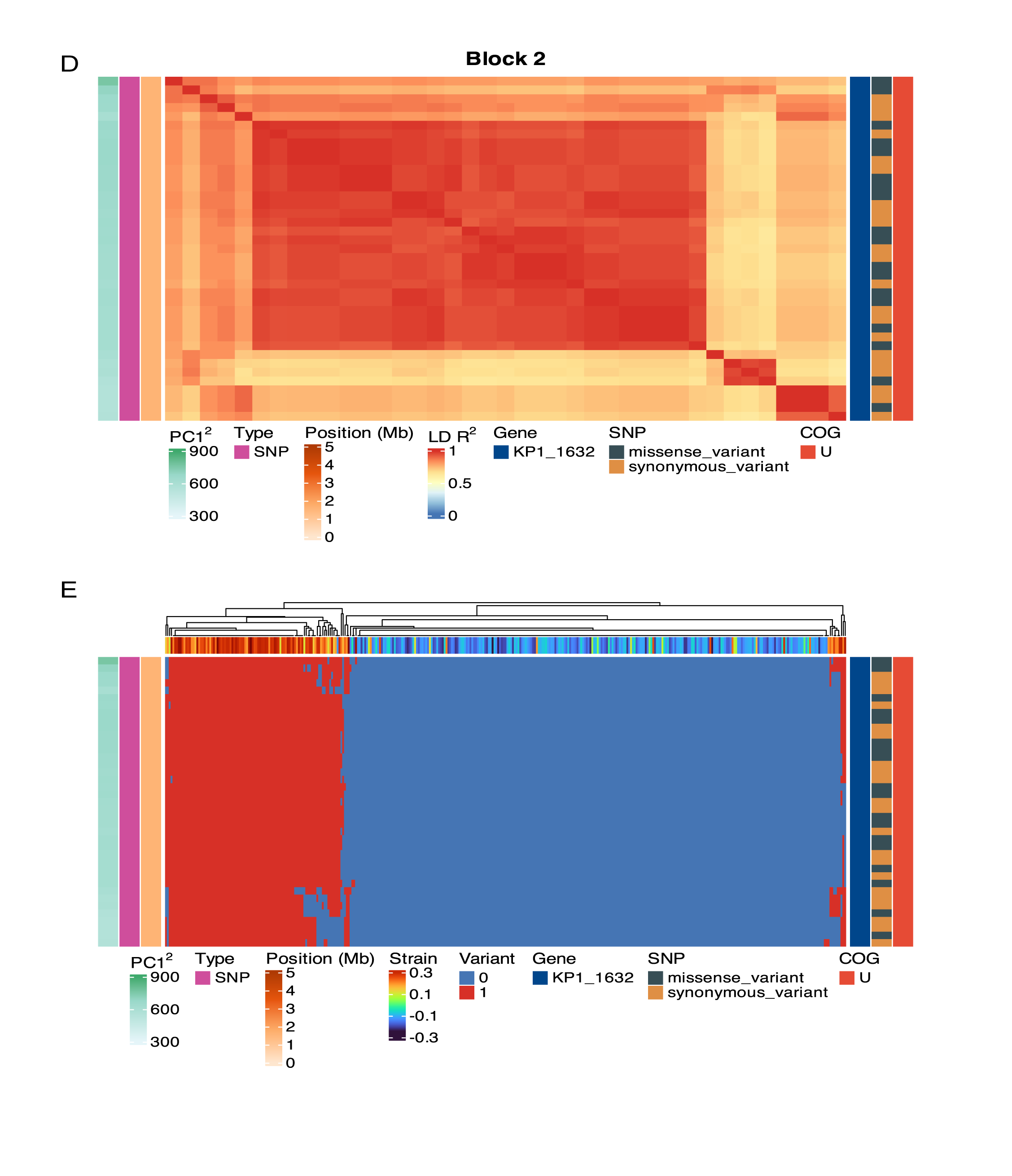

Supplement: S9 Fig — (D) High LD observed for SNPs in KP1_1632. (E) Variant (1/0) heatmap highlights distinct patterns of gene presence/absence or SNP minor/major alleles across isolates. COG one-letter code descriptions: [U] Intracellular trafficking, secretion, and vesicular transport. The data underlying this Figure can be found in https://zenodo.org/records/18520201. (TIF) [file pbio.3003672.s013.tif]

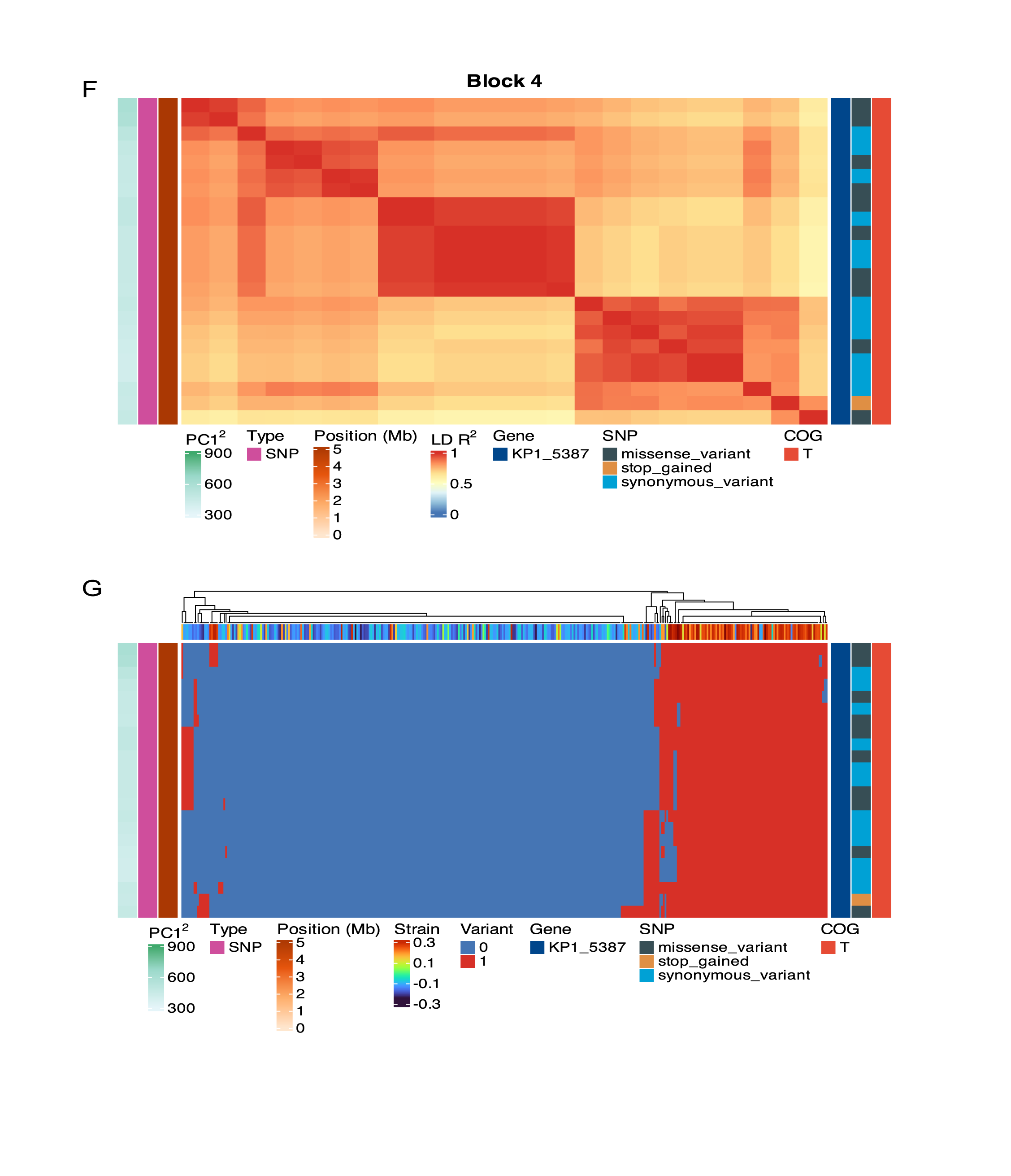

Supplement: S10 Fig — (F) High LD observed for variants in KP1_5387, forming a small, highly linked cluster. (G) Variant (1/0) heatmap shows distinct variation in gene presence/absence or SNP minor/major alleles across isolates. COG one-letter code descriptions: [T] Signal transduction mechanisms. The data underlying this Figure can be found in https://zenodo.org/records/18520201. (TIF) [file pbio.3003672.s014.tif]

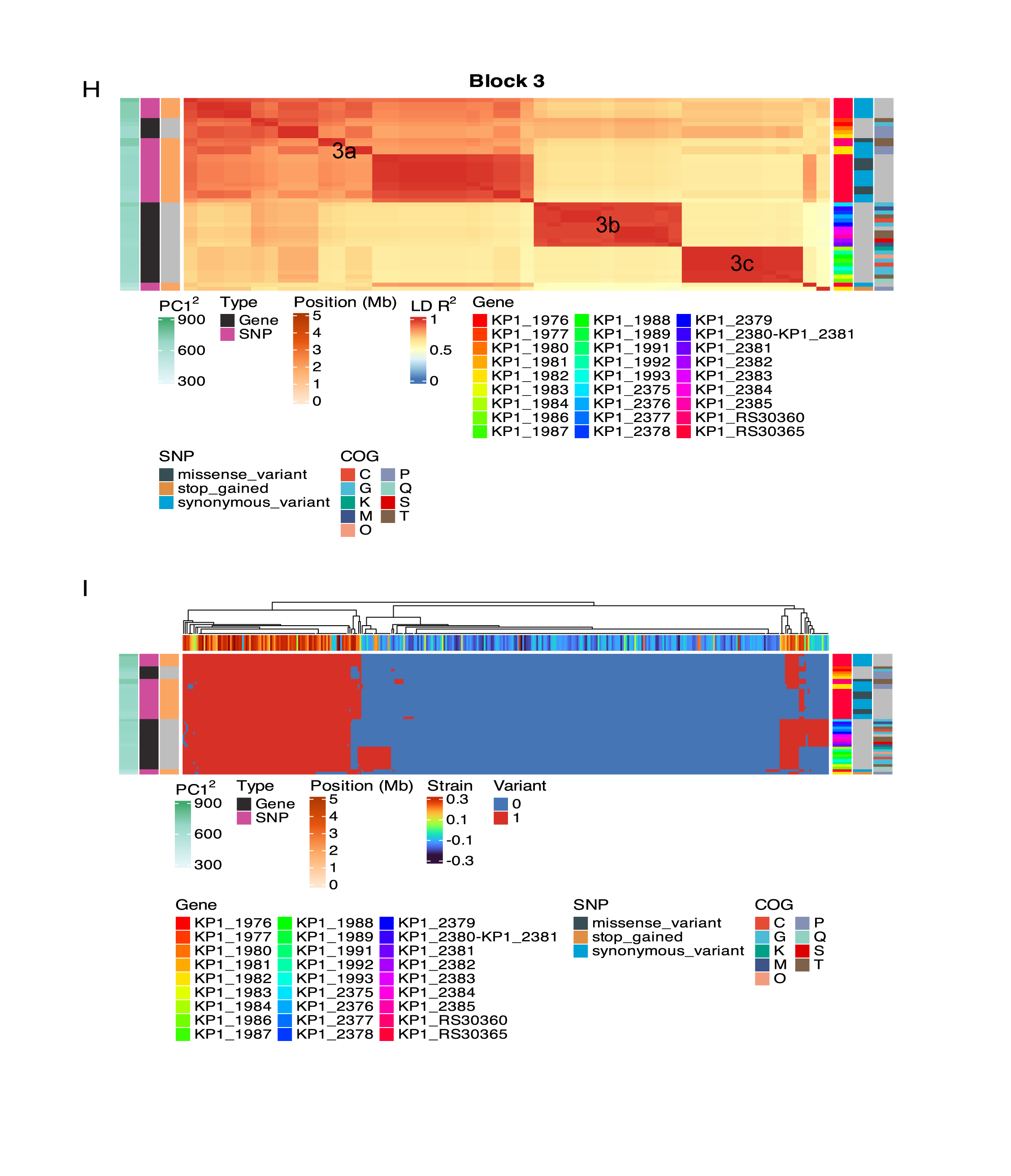

Supplement: S11 Fig — (H) SNPs in KP1_RS09345, KP1_RS30360, KP1_1982, and accessory genes form tightly linked clusters. (I) Variant (1/0) heatmap reveals clear differentiation in gene presence/absence or SNP minor/major alleles across isolates. COG one-letter code descriptions: [C] Energy production and conversion; [G] Carbohydrate transport and metabolism; [K] Transcription; [K] Transcription; [M] Cell wall/membrane/envelope biogenesis; [O] Posttranslational modification, protein turnover, chaperones; [P] Inorganic ion transport and metabolism; [Q] Secondary metabolites biosynthesis, transport and catabolism; [S] Function unknown; [T] Signal transduction mechanisms. The data underlying this Figure can be found in https://zenodo.org/records/18520201. (TIF) [file pbio.3003672.s015.tif]

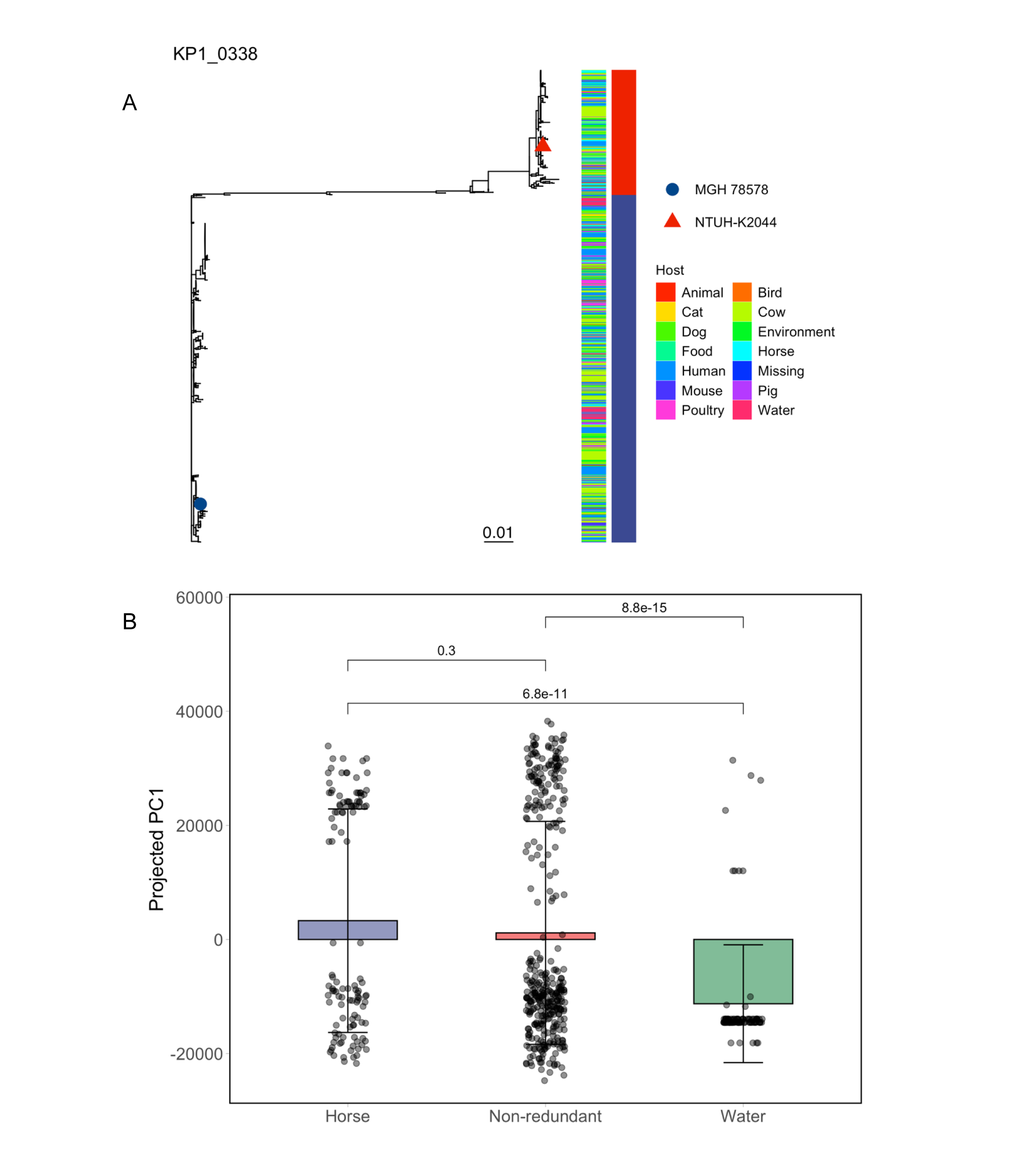

Supplement: S12 Fig — (A) Phylogenetic tree of the KP1_0338 gene from the multi-host KP dataset, with annotation strips indicating host origin and the KP1_0338 high/low classifications inferred from clustering. The canonical reference strains MGH78578 (low) and NTUH-K2044 (high) are labeled for reference. (B) Projected the multi-host dataset onto the non-redundant dataset. Each bar represents the mean PC1 value for isolates from a given host, with error bars indicating the standard deviation. The data underlying this Figure can be found in https://zenodo.org/records/18520201. (TIF) [file pbio.3003672.s016.tif]

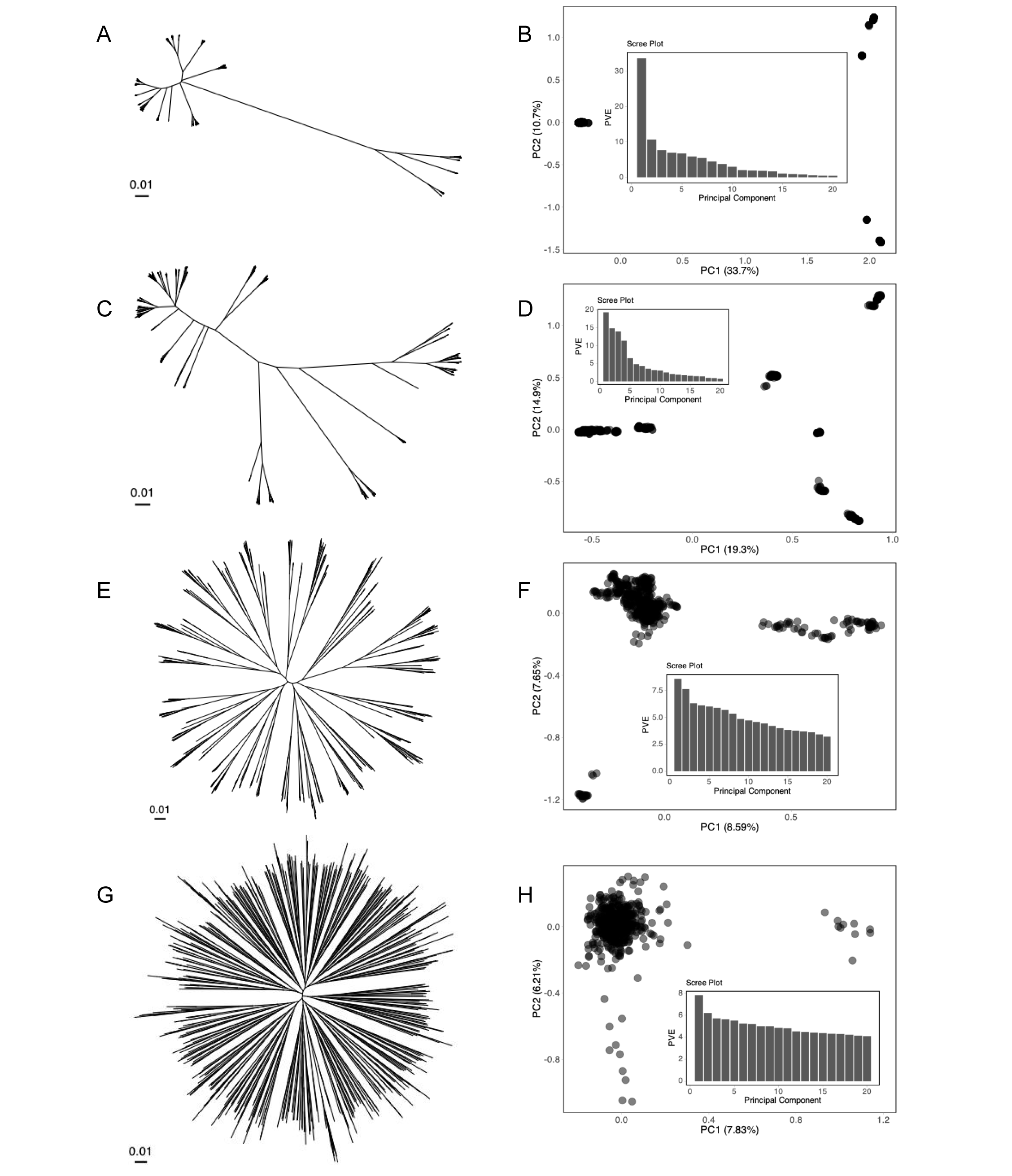

Supplement: S13 Fig — Phylogenetic trees, PCA plots, and scree plots for a population of 2000 individuals under different HGT lengths. Panels show results for: (A, B) 100 bp, (C, D) 1 kb, (E, F) 10 kb, (G, H) 100 kb. The data underlying this Figure can be found in https://zenodo.org/records/18520201. (TIF) [file pbio.3003672.s017.tif]

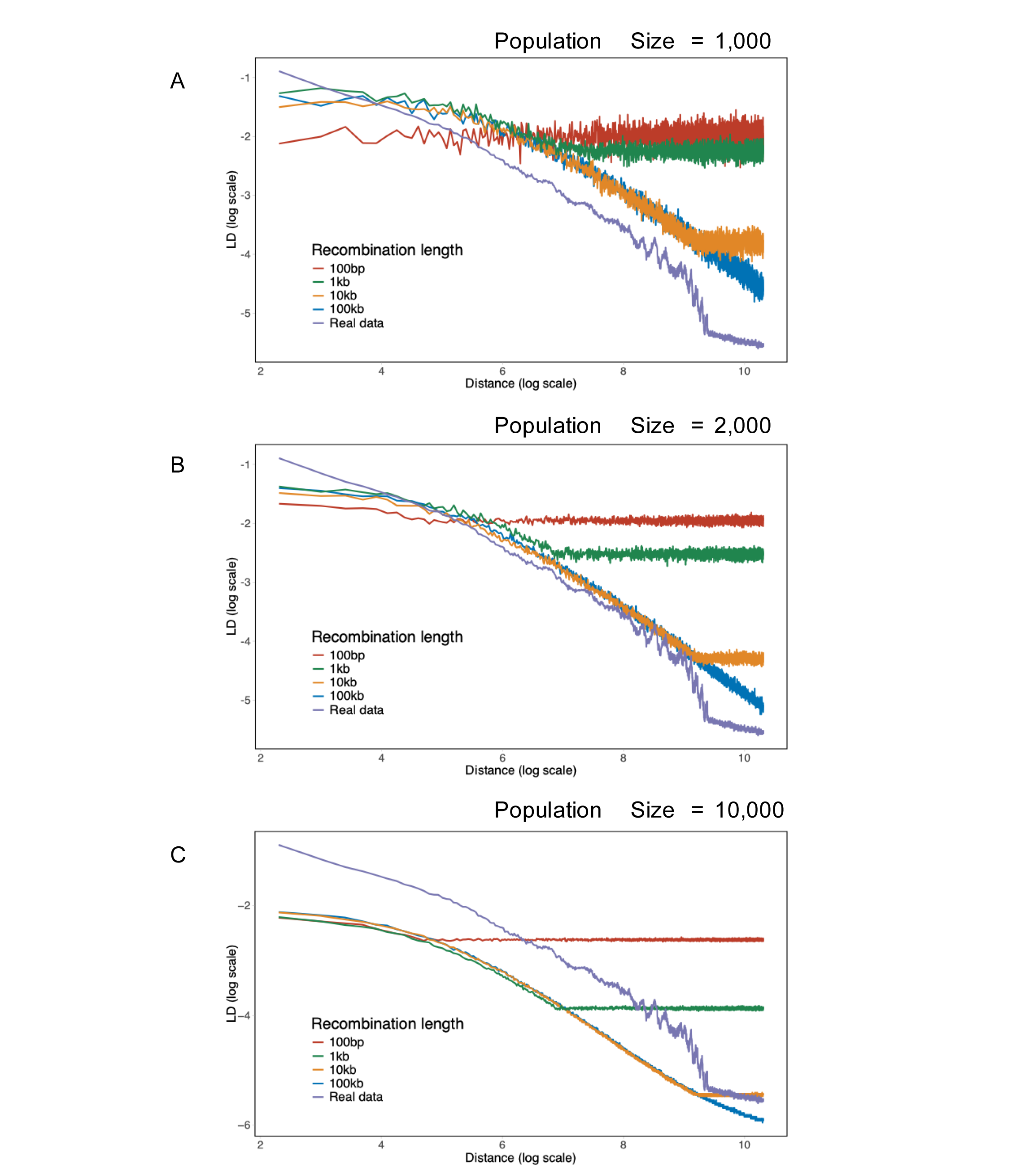

Supplement: S14 Fig — Comparison of LD decay between different HGT lengths (100, 1, 10, and 100 kb) and the non-redundant KP dataset. (A) Population size of 1,000. (B) Population size of 2,000. (C) Population size of 10,000. The x-axis represents the SNP distance (log scale), and the y-axis represents the LD value (R2) on a log scale. The data underlying this Figure can be found in https://zenodo.org/records/18520201. (TIF) [file pbio.3003672.s018.tif]

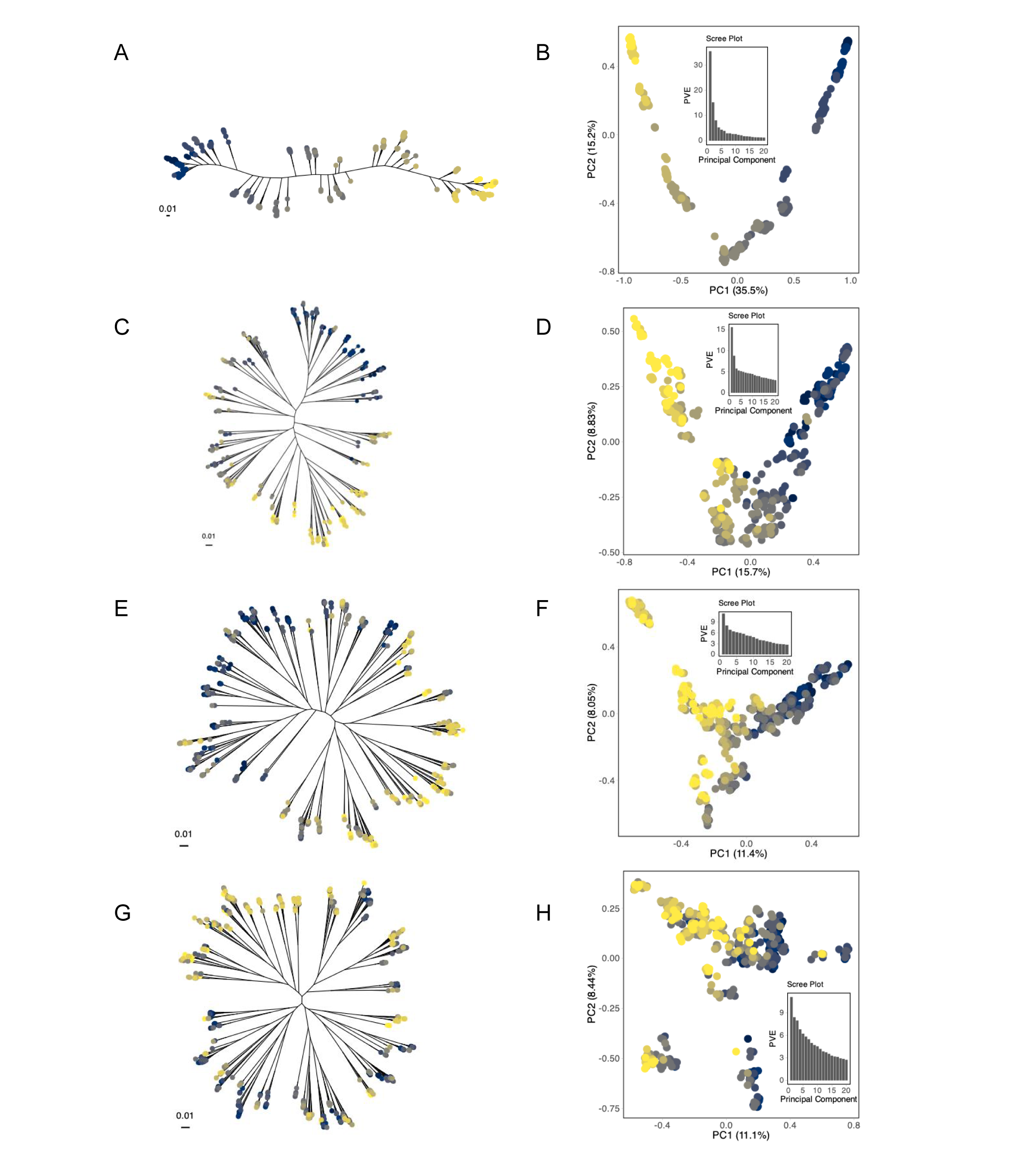

Supplement: S15 Fig — Phylogenetic trees, PCA plots, and scree plots for a model with HGT of 10 kb. The population consists of 10 subpopulations (200 individuals each). Panels show results for different numbers of migrants exchanged between neighbor subpopulations: (A, B) 1 migrant, (C, D) 10 migrants, (E, F) 20 migrants, (G, H) 50 migrants. The data underlying this Figure can be found in https://zenodo.org/records/18520201. (TIF) [file pbio.3003672.s019.tif]

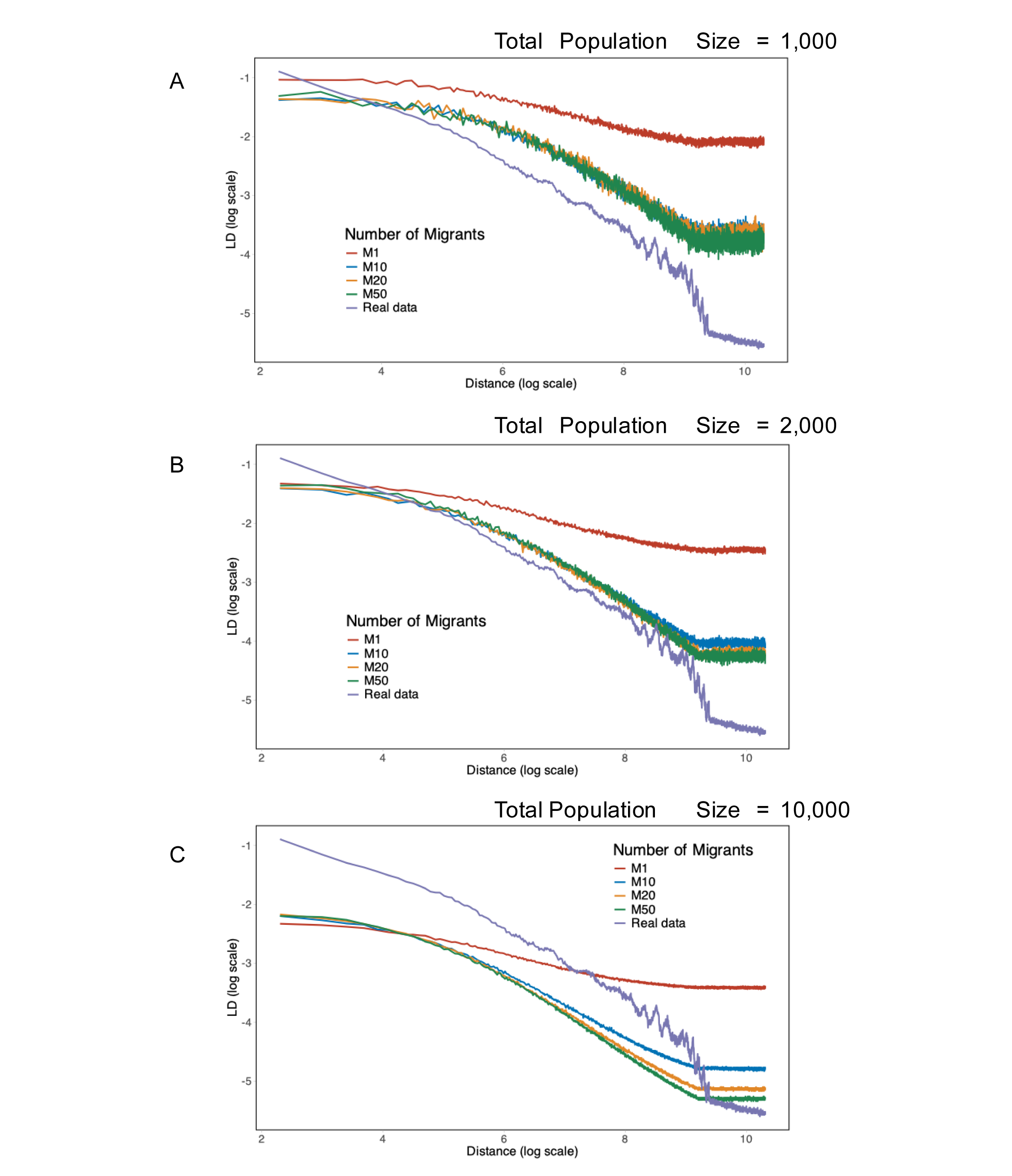

Supplement: S16 Fig — Panels illustrate results for different total population sizes: (A) 2000 individuals, (B) 10,000 individuals, (C) 100,000 individuals. Each model includes HGT of 10 kb. The x-axis represents the SNP distance (log scale), and the y-axis represents the LD value (R2) on a log scale. The data underlying this Figure can be found in https://zenodo.org/records/18520201. (TIF) [file pbio.3003672.s020.tif]

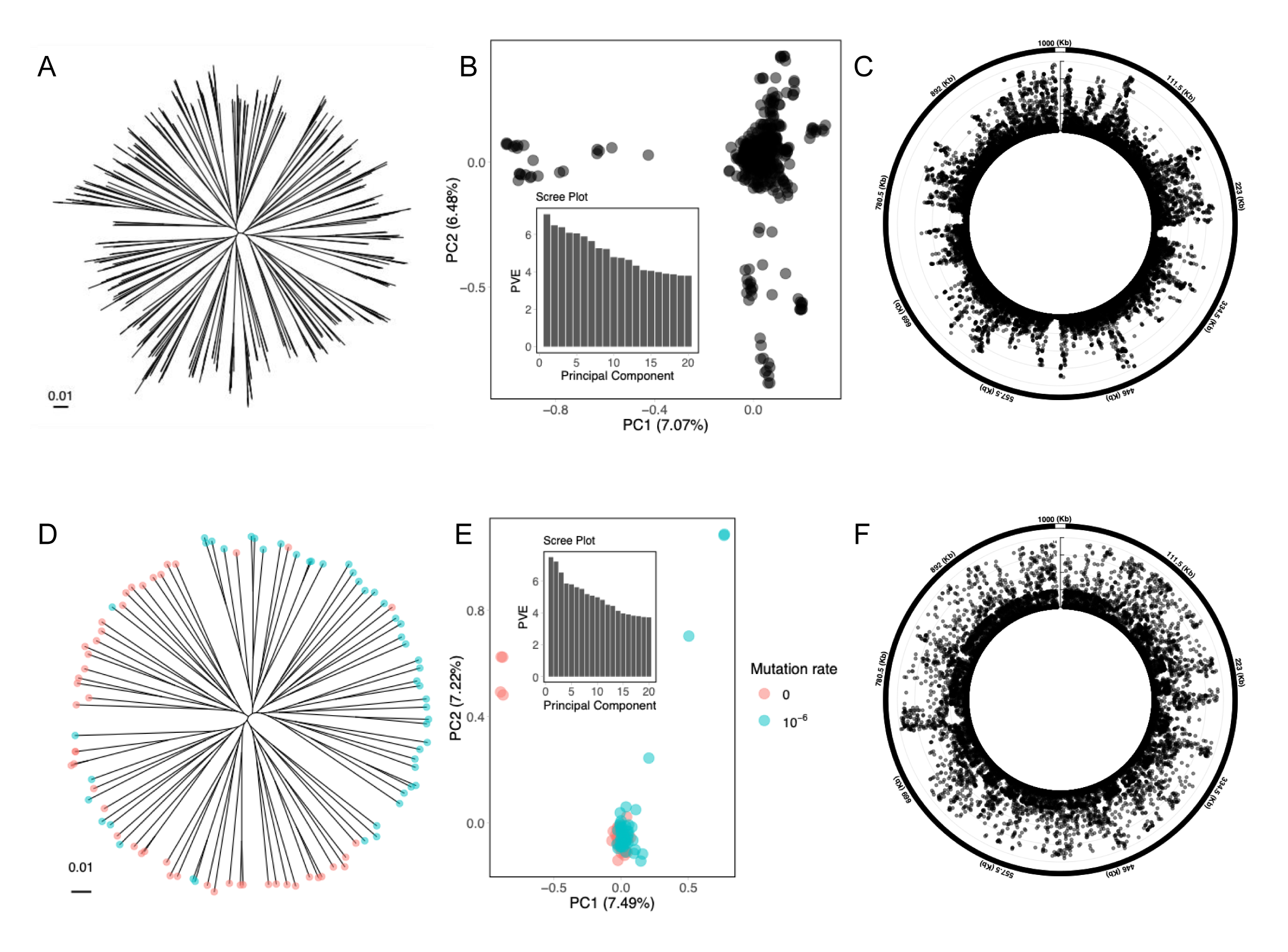

Supplement: S17 Fig — Recombination-rate heterogeneity model: (A) Maximum-likelihood phylogenetic tree, (B) PCA of the simulated genomes, (C) Circular Manhattan plot showing SNP-wise squared PC1 loadings. Regions with elevated recombination rates appear as depressions in loading values. Mutation-rate heterogeneity model: (D) Maximum-likelihood phylogenetic tree, (E) PCA of the simulated genomes, (F) Circular Manhattan plot of squared PC1 loadings. The data underlying this Figure can be found in https://zenodo.org/records/18520201. (TIF) [file pbio.3003672.s021.tif]
