# Supplementary material for: A bacterial ecocline in Klebsiella pneumoniae may explain its backboned phylogeny
Source: PLoS Biol. 2026 Mar 10;24(3):e3003672. doi: 10.1371/journal.pbio.3003672 (PMC12974863; doi:10.1371/journal.pbio.3003672)
Supplement: Attachment — Submitted filename: ReviewerResponse.docx [file pbio.3003672.s024.docx]

**Response to editorial comments**

You'll see that reviewer #1 is overall positive, but thinks that the limitations of the study should be spelled out more explicitly, and the paper should be made more accessible to the broader reader. Reviewer #2 also votes for improved accessibility, and suggests a minor analysis. Reviewer #3 is broadly positive, but thinks that you overclaim, suggesting several potential alternative suggestions (heterogeneous drift, changes in recombination rate, historical contingency…); s/he suggests a number of additional analyses and requests some clarifications. Reviewer #4, like reviewer #3, is intrigued, but not fully convinced by the strength of support. Again, more explicit consideration of alternative explanations is warranted, and there are a few extra analyses among the requests.

Thank you for these comments. We appreciate that all reviewers found that our hypothesis is new and interesting. None of the simple alternatives that we have tried, including the excellent suggestions of Reviewer #3, fit the data. In revising the paper, we have focussed on making sure our narrative is clear and consistent throughout: our manuscript describes a new and biologically interesting hypothesis which is supported by our genomic analysis but other explanations cannot be ruled out until ours is functionally validated.

**Response to Reviewers**

**Reviewer #1:**

This is an interesting and innovative study that uses the population structure and genomic diversity of *Klebsiella pneumoniae* (KP) to infer the action of selective pressure on a quantitative trait, where extreme values appear more advantageous than intermediate ones. The study relies entirely on genomic analyses (comparisons with simulations and across species), which limits its ability to identify the underlying trait under selection, leaving this open for future work. The manuscript is generally clear, and the arguments are well supported by the comparative analyses. However, it may be difficult for readers outside the field to follow, and in some cases the conclusions slightly overstate what can be inferred from the analyses. I therefore suggest that the authors moderate some statements, and add brief additional discussion or analysis of possible confounders and alternative explanations.

The study puts forward a compelling explanation for the structure of the KP phylogeny, supported by a logical set of comparative analyses (simulations and cross-species comparisons). While the authors highlight some genes strongly associated with this cline, they do not identify the trait(s) driving the structure. This is somewhat disappointing, as experimental assays could have helped explore links between quantitative traits and the observed genetic variation. That said, there is no guarantee that quantitative traits measurable in laboratory assays would capture the true driver of this subtle structuring of the global KP population. The study therefore provides evidence consistent with the existence of a quantitative trait and points to some possible functional candidates.

The argument that selection on a quantitative trait could explain this structure is convincing, though alternative explanations have not been fully excluded. This is inherently difficult, since simulations only capture simplified scenarios, and the dataset is affected by inevitable sampling biases. The authors aim to explain the 'backbone' structure, the overrepresentation of samples at its extremes, and the uneven genomic associations with the backbone. Taken together, these features point towards diversifying selection on a quantitative trait as a likely driver. Nonetheless, given the complexity of bacterial genome evolution and population structure, unknown aspects of evolutionary history and ecology, and the potential for sampling biases, some caution is warranted.

Thank you for the positive comments. We agree that our explanation is innovative and compelling. We also wholeheartedly agree with the reviewer that caution is warranted, and we try to make this clear from the outset, which is why the second word of the title is *putative* (line 1). Our abstract also makes it clear that it is a hypothesis that needs to be validated by identifying the trait *if it exists* (line 24). We have added a further disclaimer in the introduction “*if this explanation is correct*” (line 53) and in the discussion (particularly lines 566-571).

The authors explore possible links between metadata and population structure (geography, virulence, resistance), but they might also consider other confounders such as sampling date, host source, genome size, and base composition. While they touch on host and environmental sources, and their connection to geography, this could be discussed earlier as a possible driver of the patterns observed. They might also reflect on potential (including unrecognised) sources of sampling bias. For example, if all isolates were cultured before sequencing, the growth conditions themselves may have imposed selective pressures.

We do not think that sampling bias is a likely cause of the structure that is observed because the structure is deep in the tree and for this species the global database is geographically and ecologically diverse (see for example citations 11-13). Geographical differentiation is difficult to completely rule out but in common with other enteric bacteria *Klebsiella* is dispersed readily by birds and shows no sign of differentiation at any geographical resolution.

It would also be helpful if the discussion acknowledged how more complex scenarios could generate the observed patterns. For instance, while geographic isolation alone may explain the backbone but not other features, could combining isolation with restricted recombination (e.g. isolation by distance) do so? Could the apparent lack of clear geographic structure reflect reduced geographic differentiation in recent years due to greater movement of humans and livestock? In addition, the simulations do not include the accumulation of variation through interspecies recombination (which the authors observe in their block 1). Could the combination of geographic structure and constraints on recombination explain the observed patterns? While it would be unreasonable to expect the authors to model all such scenarios, it would strengthen the manuscript to either explain why these more complex alternatives are unlikely explanation of the observed patterns, or acknowledge that they remain possible.

We now explicitly acknowledge in the discussion that these more complex explanations remain possible (especially lines 613-617).

Although clearly written overall, the manuscript is likely to be challenging for non-specialists in population genetics or genomics. Adding more explanation of the reasoning behind the analyses would make the work more accessible, particularly for readers from other disciplines who might build on these findings through experimental or observational work (as the authors suggest through a 'reverse ecology' approach).

See response to the same point made by reviewer 2 below.

Finally, there are a few places where the strength of the conclusions is overstated, particularly where no clear threshold distinguishes presence or absence of a given pattern. For example, in lines 124-125 the authors claim that they can 'falsify the simplest neutral model' based on their results. This goes beyond what can be concluded: no formal test is presented, and absence of evidence (of similar structure) does not amount to evidence of absence.

We have reworded this section (lines 132-135).

**Reviewer #2:**

This study addresses the population genetic structure of *Klebsiella pneumoniae* (KP), a species known for global dipersal and high recombination rates. Phylogenies generated here reveal a backbone-like structure, corresponding to a major axis of variation (PC1) that explains 16.8% of genetic diversity. The authors propose that this reflects a "bacterial ecocline" shaped by diversifying selection on a quantitative trait rather than solely demographic processes. Using simulations they demonstrate which parameters can reproduce observed KP genetic patterns. The association of PC1 with loci including Kpa fimbriae genes, suggests modulation of adhesive properties could be driving the evolution. This hypothesis was not empirically proven, but that seems outside the scope of the study.

Major comments:

1. As a reviewer outside of evolutionary biology, I had a difficult time understanding some of the language used. To make this article more broadly accessible and, thus, more impactful, I suggest providing more explanation of the jargony terms. Some examples include "PC1 loadings", "absolute PC1 loading values", "LD decay curve", "trait value density", "Gamma distribution", etc.

We agree with the reviewer that the manuscript should be as accessible as possible. We have revised the text to clarify the technical terms mentioned, including “PC1 loadings,” “absolute PC1 loading values,” “LD decay curve,” “trait value density,” and “Gamma distribution.” For each of these terms, we provide more relevant context in the main text and added a brief explanation at first mention in the **methods section**. These revisions aim to improve readability and ensure that the manuscript is understandable to a broader audience.

2. It seems like the 4 SNP sites are all distributed within 2.5 Mb of each other. Where do the 4 SNP enrichment sites (Fig 4A) fall on the DNA schematic in 2C? Are the 4 sites split evenly between left-hand and right-hand groups? If they were not split evenly between left and right side, I wonder if Fig 2 is repeated with the 4 SNP sites weighted on the left-side and no SNP sites fall on the right side, if the ecocline would be lost on the right side. This could strengthen the conclusion that the 4 SNP sites are driving the observed backbone.

We thank the reviewer for this helpful comment. The four SNP enrichment peaks identified in Fig. 4A are not all located on the same side of the chromosome. The first three peaks are clustered within a 2.5 Mb region, while the fourth peak is positioned on the opposite half of the chromosome. These sites are now indicated on the DNA schematic in Fig. 2C. This distribution shows that the enriched SNPs are not restricted to one side of the backbone structure, suggesting that the ecocline pattern is not solely driven by variation in a single genomic region.

Minor comments:

3. Generally figure axis labels and figure-embedded text are small and difficult to read. Also, it is difficult to discriminate the blue trace of 'real data' vs the blue traces of some simulated data (throughout manuscript). Perhaps switch 'real data' to black, green, or red traces so it is clearly distinguished from the simulated data. Or use a heavier weight line.

Thank you yes. We have retained the same color scheme to ensure consistency across all figures but have increased the line thickness and contrast of the real data trace to make it clearly distinguishable from the simulated data. In addition, the figure legends now explicitly specify which line corresponds to the real data. We believe these adjustments significantly improve clarity while maintaining a consistent visual presentation throughout the manuscript.

4. Fig 3 section references "Molassodon" ecospecies in the text, but does not identify them in Fig 3. Is Eg1a Molassodon ecospecies. If not, what is Eg1a vs non-Eg1a?

We thank the reviewer for pointing out this potential source of confusion. The EG1a ecogroup is a designation used in Reference 23 which however obscures its distinctive nature as an ecospecies. For this reason, it was renamed as the Molassodon ecospecies in Reference 28, as is now explained in the text (lines 140-144) and we now use this name elsewhere.

Line 152 typo - "dataset of," the dataset is not defined.

The sentence has been corrected to read “PCA of the dataset of VppAsia, using the same legend as in panel g.” (line 163-163)

5. I believe all genome loci are referred to using NTUH-K2044 locus tags. It would be helpful to explicitly state that early in the Fig 4 data section. Perhaps report that in Fig 4 and Fig S6 legends.

We thank the reviewer for this helpful suggestion. All genomic loci are indeed referred to using NTUH-K2044 locus tags. We have now explicitly added corresponding notes to the legend of Fig. 4 (line 170) to clarify the locus tag reference system.

6. Cross-check the results text and Supplementary Fig 7 legend. It appears that panel orders were switched and some Fig S7 panels are not identified accurately.

Oops sorry! We have carefully cross-checked the Results text and the legend for S7 Fig. The panel order and labels have now been corrected to match the figure layout, and all panel references in the text have been updated accordingly (line 1317-1342).

7. Line 232 - I think 'solid (a) and dashed (b)' should be switched to 'dashed (a) and solid (b)'

Good point! The description has been corrected to read “dashed (A) and solid (B)” as suggested (line 247).

8. Would it be possible to write COG letters in Fig 5B and 5C? This could help readers link COG letters embedded in Fig S7. Alternatively, could the function of the COG letters in Fig S7 be provided in the legends?

Good suggestion! We have added the function of the COG letters in S7 Fig legend (line 1317-1342). These revisions improve the connection between the main and supplementary figures and enhance interpretability.

9. Fig S8 - States that panel A show KP1_0338 high or low, but MGH vs NTUH is just identified. It would help reader comprehension if the KP1_0338 high and low strains are identified alongside their name in the figure panel.

Thank you for the helpful suggestion. In Supplementary Fig. S8A, our aim was to illustrate the contrast between the KP1_0338–high and KP1_0338–low groups using the two best-characterized reference genomes, MGH78578 and NTUH-K2044. These strains represent canonical high and low states, respectively, and are widely used as reference genomes in *K. pneumoniae* research. For this reason, they were specifically highlighted in the figure, whereas annotating all strains in each group would not further improve clarity. To address the reviewer’s comment, we have now updated the figure caption to explicitly indicate that MGH78578 is KP1_0338–high and NTUH-K2044 is KP1_0338–low (line 1344-1347). This improves readability while preserving the intended illustrative purpose of the panel.

**Reviewer #3:**

This paper explores why there is a "backbone" -- a connected path of relatively long internal branches -- in phylogenies of *K. pneumo*.

The authors argue that such a pattern is not observed in simple neutral models and is likely due to an "ecocline": a quantitative trait under diversifying selection.

I really appreciate some aspects of this: the big-picture view and interest in a fundamental evolutionary question; the use of the tree shape; the use of simulations to help explore what-if scenarios (rather than, say, simply assuming that a model such as one with diversifying selection must have given rise to the data, then estimating a parameter in that model, then believing that estimate). However, there are some major limitations and a few instances where I think the authors have over-claimed.

The scientific argument is that diversifying selection on a quantitative trait can produce an observed pattern, and maybe that it is plausible (though the trait is yet to be identified). It is hard to argue that it is the only thing that could plausibly produce the pattern, or, to even characterize what else might be able to produce the same pattern. What happens in the model if there is no selection, just drift, but with a heterogenous distribution of drift rates across the genome? What about changes in the recombination rate over time, with deep branches not having much, and then more movement? would historical contingency create an apparent backbone?

In order of appearance in the manuscript (not importance):

How much phylogenetic support is there for the deep branches that define the "backbone"?

We are reluctant to get too deeply into interpreting phylogenetic results because we think that while universally used to visualize and interpret relationships, phylogenetics is fundamentally a wrong model for studying evolutionary processes in highly recombinant bacterial populations. We have rewritten key parts of the results to emphasize this key point (e.g. line 60-65). Most pertinently, under a model of a continuously varying trait, we do not expect any specific deep branch to be well-supported. The characteristic backbone feature is rather that there is just a single path connecting the internal branches of the tree (see response below) and that there is some consistency in signal between them, which is what the PCA picks up

Phylo reconstruction algorithms are known to produce "ladder shapes" where there isn't much support for a different branching pattern; there is long branch attraction, etc. The fact that the same backbone appears in different trees is reasonable evidence but you could try raxml or iqtree especially where you have only 100s of tips, not 1000s. Even for 1000s these are very feasible.

We thank the reviewer for this suggestion. Although we are cautious about over-interpreting phylogenetic trees in highly recombinant bacterial populations, we agree that it is important to confirm that the backbone pattern is not an artifact of a specific algorithm. We therefore re-inferred the phylogeny of the non-redundant dataset using maximum likelihood in IQ-TREE. The resulting topology is highly similar to the FastTree reconstruction and shows the same backbone structure (S2 Fig. A).

This consistency across algorithms indicates that the backbone pattern we describe does not arise from ladderization or long-branch attraction, but reflects a robust feature of the data rather than a method-specific artifact.

Phylogenetically, the backbone is a connected path consisting of relatively long internal branches, meaning that they are longer by quite a bit than other internal branches. And it's the only such path, and only branches in a certain length range are in it (pretty much). Does this define the backbone?

This is a very nice description of what the backbone is – and we have inserted it into the text (line 78-79). Thank you so much!

They are shorter than the pendant branches and consistently longer than all other internal branches. You could quantify this property rather than relying on the layout to define it (since tree layout algorithms can be very misleading). That way, you could also see to what extent it appears in simulations, without relying on visualizing trees one by one to see whether a backbone is emerging. You could define a backbone in terms of the number of (connected) longer internal branches, their depth in the tree, and their relative length (compared to the other internal branches and compared to pendant branches).

We agree that it would be possible to do this, however as we now argue more explicitly, we think that PCA is intrinsically more suitable for describing continuously varying traits. The key point, shown in Figure 1, is that the backbone and PC1 results are highly congruent, suggesting that despite very different assumptions, the two methods both capture the same strong signal.

Line 104 - re left/right split and clonal structure: no, the right and left parts of the tree aren't the clonal structure that the tree captures (such as it is), because there is no split in the tree corresponding to left/right. In fact the PCA *is* connected to the clonal structure, as you can see in figure 1 with the colours. But because the clonal structure is weak, the pca axes capture little of the variation and aren't too connected to the tree. Suggest removing the artificial left-right split. It's also very dependent on the layout algorithm.

Thank you for the comment. It is true that there is no deep left right split in the tree, and therefore which of the deeper splits cluster together is dependent on the layout algorithm and thus essentially random. However, what this clustering achieves is that sets of strains which do have some residual phylogenetic signal s*hallower in the tree than the backbone* cluster together on one side or other. Therefore, the fact that left and right give similar results show that the signal is not dependent on the residual phylogenetic signal within any particular “deep clone”. The fact that PC1 does correlate well with the backbone, while not being related to any of the shallower branches is the main point here. We have added a sentence of explanation to make this clear (line 112-114).

p5 line 125 this seems like an overclaim - you can't falsify a whole model class based on a few simulations and a PCA. Fitting the model parameters might allow you to capture the observed patterns (which anyway you haven't quantified).

This section has been reworded.

p7 line 163 onwards I was losing the story a bit. Why are these results about genes and their loading on PCA1 and their LD here? Why not instead look at the splits of the taxa that are indicated by the edges defining the backbone? These would be the most direct questions to suggest what the trait might be, if there is one, because whatever the gene/ SNP differences are along the backbone describes the evolution that defines the backbone. If there is strong phylogenetic support for those edges, there is presumably also support for the changes that those edges define.

Thank you for the suggestion. We hope that our rewording (especially lines 60-65, lines 75-79 and lines 90-97) makes our logic clear. An informal count suggests that the backbone consists of around 12 internal branches of similar-ish length, which would mean 12 lists of candidate genes. Whether or not any of these is strongly supported, the overall PC1 signal averages across all 12 and hence is a better basis for defining the putative underlying quantitative trait. Another way to explain this is that to identify the basis of the trait by phenotyping, it is a good idea to start with strains at both ends, which are predicted to have the biggest differences in trait values. PC1 and phylogenetics are both methods for capturing genome-wide patterns of non random-association (or linkage disequilibrium) but PC1 is more suitable to capturing this particular form of LD, since the fitting implicitly assumes continuous values with extremes, while phylogenetics implicitly assumes that the trait takes discrete independently varying values on each branch.

L188 what does "encoding the usher" mean?

“Encoding the usher’’ refers to the fact that *kpaC* encodes the outer-membrane usher protein of the chaperone–usher fimbrial assembly pathway. We have revised the text to make this clearer (line 203-204).

page 10 -- i don't know where the approximately causal language comes from. PCA vectors are defined by the data. They don't have "influence". They aren't, in this case, even population structure. Again (see previous comment) I wondered about the long branches in the backbone; would it not be more interesting to look at which mutations are different from top to bottom of this backbone? How similar would this be to the PCA? judging from fig 1a there would be some similarity, but they are not the same.

Thank you for this comment. We have removed the causal wording. Our intention was simply to say that PC1 captures the main axis of variation in the dataset, which corresponds to the backbone structure.

We used PC1 loadings because PC1 provides a compact summary of the genetic differences along this axis and have better support and are more interpretable than any of the dozen or so internal branches considered individually.

Line 247 re virulence, AMR, hosts, etc: did you test for phylogenetic signal ("clustering")? can we see the trees with the colours corresponding to virulence, amr, geography, host, etc?

Thank you for the suggestion. We did not perform a formal phylogenetic signal test, but we examined the distributions of virulence, AMR, host and geographic categories. These traits do not show clear clustering along the backbone or within the major groups.

Because PC1 reflects the backbone structure, plotting these variables on the PCA gives the same information as colouring the phylogenetic tree, but in a simpler and more interpretable way. We have clarified this point in the text (line 86-88).

It wouldn't surprise me if virulence (in humans) doesn't matter; why would it (except for sampling artifacts, several of which you go on to describe clearly). Anyway, visually not observing clustering (and not even showing it) isn't strong evidence; there can be phylogenetic signal that isn't immediately apparent visually.

Thank you for this insightful comment. We agree that there is no strong biological reason to expect human virulence to exhibit a clear phylogenetic pattern, aside from possible sampling artifacts. We also agree that the absence of visually obvious clustering does not rule out the presence of phylogenetic signal, which may be subtle or not apparent from inspection alone.

To address this point, we included a dedicated section in the main text from line 256 (“Virulence, antibiotic and isolation source profiles provide few clues”) where we summarise why these traits show little correspondence with the major splits of the tree. The purpose of that section is not to claim that phylogenetic signal is absent, but to emphasise that these traits provide limited evidence for strong structuring along the backbone.

page 12 -actually supplementary fig 9g is getting there! i think I see a backbone.

what happens as you change other parameters, like N?

Thank you for these comments. While Supplementary Fig. 9g shows some variation in internal branch lengths, the longer branches occur in separate parts of the tree and do not form a continuous series, so we do not regard this as a backbone.

Changing other parameters, including the population size N, has little effect on the qualitative tree structure; it mainly influences LD decay. For this reason, we show the impact of varying N on LD in the Supplementary Materials (S10 Fig and S12 Fig).

More broadly, why are the parameters you choose for the simulations very likely to be the best ones for trying to find the effect you are trying to find? (Likely it is hard to know a priori, but this means that trying a few out and not seeing the phenomenon you are interested in is quite weak evidence).

Thank you for raising this point. Our aim was not to identify a precise set of parameters for which the neutral model fails, but to check whether a range of simple and biologically reasonable settings could generate a backbone-like pattern. The parameters we used fall within values that recapitulate the basic patterns of diversity in KP (i.e. we chose population size, mutation rate and HGT rate to give diversity levels and LD levels similar to KP), and they span the ranges that would be expected to affect the depth and shape of the tree.

Across these different settings, the simulations varied in overall branch lengths but did not produce a continuous series of long internal branches. This suggests that the absence of a backbone-like structure is not specific to a particular choice of parameters. Our conclusions therefore rely on the robustness of this qualitative result rather than on the assumption that we have identified a single “best” parameter set.

Without a quantitative description of the phenomenon you are interested in (see above comment) you can’t assess to what extent it may be emerging in simulations.

What about: heritable high substitution rate that is then lost, but whose effects remain, deep in the tree?

A lot is made of the visual appearance of the tree. Even if you want to stick to visual comparisons, you could look at what the tree looks like in a rooted view? With the root at one end of the backbone,? With the root in the middle? Etc. Rooted trees can suggest different interpretations, which might be interesting.

We agree that the visual layout of a tree is not a unique or decisive criterion for interpretation. In our analysis, the tree visualization is not used in isolation, but rather as a qualitative representation that is consistent with the primary signal captured by PC1. We additionally examined the tree under alternative rootings. These rooted views do not alter the underlying backbone structure and remain fully consistent with the PC1-based interpretation.

Overall I think it’s interesting. But having done a lot of modelling, I would be very cautious in simulating something, seeing it not match the data, and then concluding that the model *cannot* match the data. There is usually a **lot** of work between “this didn’t look like the data when I simulated with my first guess at the parameters” and “this model *cannot* generate a pattern like the one seen in data”.

Our intention was not to claim that the alternative models are fundamentally incapable of generating the observed structure and have restructured the manuscript to emphasize that we are proposing a new explanation, not seeking to prove that it is the only one, as described in the response to all reviewers above.

Line 596: iterative popPUNK selection: what does this mean? Please say more about what an isolate needs to do to get into the data. What sampling (tended to) result in inclusion in the poppunk or kleborate samples?

We thank the reviewer for pointing out that this phrasing was unclear. We did not perform any iterative selection of isolates. The dataset referred to here is simply the publicly available PopPUNK test dataset provided by the original authors, which we used without any additional filtering. The term “iterative selection” has now been removed to avoid confusion, and we have clarified that these isolates come directly from the PopPUNK reference dataset (line 640-641).

I don't work with this organism but if you have core SNPs in 99% of the isolates doesn't that mean you have a poor choice of reference?

We thank the reviewer for raising this point. The use of “99% of isolates” does not reflect incomplete mapping to the reference genome. Instead, it follows the standard definition of core genes and core SNPs in pangenome analysis, where a gene (or aligned position) is considered “core” if it is present in ≥99% of isolates. This threshold is widely used by tools such as Roary and Panaroo to account for occasional missing data or assembly gaps. Therefore, the choice of reference genome does not affect this definition.

Line 613 fix grammar.

Fixed

What's R2 in haploview? some explanation for those not familiar with it would help.

We now clarify in the Methods that Haploview reports LD using *R²*, the squared correlation coefficient between alleles at two loci, which is a standard measure of linkage disequilibrium strength.

line 756 Isolation → isolate?

Oops! Corrected.

With recombination rates - line 835 - what is the interpretation of "generation"? presumably even KP doesn't get recombination opportunities, never mind successes, on every cell division? what happens to the simulated data and its ecocline (or lack of) as you change simulation parameters?

In our simulations, each cell is assigned one recombination event per generation. This was not intended to represent the literal per-division HGT frequency in KP, but rather follows a standard rate-rescaling strategy used in forward population-genetic simulations of highly recombining bacteria.

Our initial simulations used a 1 Mb genome, which is substantially smaller than the 5 Mb KP chromosome. To maintain a realistic effective genome-wide recombination rate and to allow the model to reach equilibrium within feasible computational time, we increased the per-generation HGT rate. We later added simulations with a 5 Mb genome that more closely approximates the real genome size; in these, we retained the same modeling framework, as SLiM implements recombination as a simplified per-generation event rather than modeling the full mechanistic complexity of HGT.

**Reviewer #4:**

In this manuscript, Liu et al., first analyse the population structure of *Klebsiella pneumoniae* (KP), describing a phylogenetic structure with lineages radiating from a backbone. They then propose a model in which diversifying selection on a quantitative trait reproduces this structure. The authors hypothesise the backbone therefore reflects a bacterial ecocline.

I found this work very interesting and the insights valuable - particularly in introducing quantitative traits in our thinking about bacterial evolution. The overall approach is convincing, though I do not have the right expertise for an in-depth technical review of the genomic analyses. Given its complexity, the manuscript is clearly written. I did not find the evidence for the ecocline model entirely convincing (in particular because of how alternative explanations were treated - see below), however I do not think this is a problem: the authors are very clear this model is presented as a hypothesis.

In my view, this work is a good fit for Plos Biology, but I do have a few points that I think are very important to address and some minor suggestions.

Essential points:

1. I was not able to find any details on the genomic data (other than most of them being from humans, line 250), which makes interpreting the genomic analyses more challenging. At a minimum, it would be helpful to have the total number of isolates, and the distribution of isolates from different host types and different geographical locations. If the information is available, it would also be very helpful to know whether human and environmental isolates are hospital-associated or from the community.

We have added a detailed description of the genomic dataset in the Methods section and clarified that the full metadata are available in Supplementary Table 2.

2. For all of the modelling results, it was unclear to me whether the 10k generations represent an equilibrium state. Are the results (e.g. shape of the phylogenies, presence of intermediate trait values) robust to running the simulations for longer? In particular, it was not clear from the results as presented here whether the model predicts a stable phylogenetic backbone, or whether this is a temporary feature and simulations eventually converge to either two distinct populations or a population with no structure.

Thank you for the question. We tested neutral simulations run for 5,000, 10,000, and 20,000 generations, and found no qualitative differences in tree shape or the distribution of intermediate trait values. Longer runs did not produce a stable backbone or cause convergence to either two populations or a fully unstructured population. Because results were robust and 10,000 generations is commonly used in SLiM studies, we adopted this duration.

3. I did not entirely follow the reasoning for dismissing heterogeneity in recombination rates between strains / ecological demes as an explanation for the phylogenetic backbone.

The authors exclude heterogeneity in recombination rates in part because this reduces the effectiveness of recombination" and therefore likely makes it difficult to recapitulate the low levels of LD in KP at large genetic distances (line 296). I do not follow the logic here, one could also frame these models as increasing the effectiveness of recombination in closely related strains relative to distantly related species. More generally, the authors parameterise recombination based on fitting the LD decay curve - it would seem surprising if including heterogeneity in recombination rate (which increases the dimensionality of the model), would make it difficult to fit this curve. I understand the authors also dismiss this explanation based on previous results, however, the argument would be considerably stronger if this alternative model were explicitly explored.

I understood the reasoning for why the geographical model is an unlikely explanation for the data, as there is no evidence of geographical structuring, but not why this structuring could not arise from ecological demes (line 329). If the analysed genomes are primarily from humans, the demes would have to exist within the human population (e.g. age structure or hospital vs community), so the argument about acquisition of KP by humans from other sources does not seem relevant.

Overall, I think the manuscript would benefit from a clearer discussion of the ecocline hypothesis in relation to alternative hypotheses and ideally from additional simulations of alternative models, particularly relating to heterogeneity in recombination rate.

Thank you for raising these points. We agree that heterogeneity in recombination rate is a plausible alternative hypothesis and have therefore now examined it explicitly. We implemented two forms of heterogeneity: (i) strong genomic recombination hotspots and (ii) persistent individual-level variation in mutation rate, which creates variation in effective recombination rate without introducing population structure. Neither model generated a backbone-like phylogeny, PC separation, or LD decay resembling the real KP data, and hotspots in particular tended to homogenize the population rather than produce deep splits (new results shown in Fig. S13). Thus, introducing recombination-rate heterogeneity does not improve the fit and cannot account for the observed patterns.

Regarding ecological demes, we note that the backbone pattern is absent from trait categories that could plausibly define within-human demes (e.g. virulence, AMR, isolation source). The lack of correspondence between these traits and the major splits argues against an unobserved ecological subdivision within the sampled human population.

Minor points:

Line 243: "our analysis suggests that they are not the primary determinants of its overall population structure, which is not unexpected given that most lineages only cause human disease sporadically." This is not relevant in the context of antibiotic resistance, as KP is also exposed to antibiotics when carried asymptomatically ("bystander selection").

We agree that KP may experience antibiotic exposure outside clinical infections. However, our point was not to imply limited antibiotic exposure, but rather to highlight the ecological context of KP as a widespread environmental and opportunistic species. We have therefore retained the statement that most lineages only cause human disease sporadically and have added a clarifying sentence emphasizing the species’ environmental and commensal distribution (line 260-262).

Line 847: In the model, the average number of offspring is >1. Is the population therefore growing, or is there an additional step to maintain constant population size?

In our SLiM model, the population size was fixed at 10,000 individuals (parameter K). Although the number of offspring per individual was drawn from a Poisson distribution with a mean of 2, excess offspring were removed through density-dependent regulation to maintain the constant population size.

Supplementary table 1: I thought most of the isolates were from humans, but this is missing in the table.

S1 Table indeed excludes human isolates by design, as it was specifically generated for the host-shift analysis described in the Methods section. The goal of this subset was to focus on non-human sources to examine host-associated patterns without the strong bias from human-derived isolates.

Some of the figures are quite difficult to read - e.g. very small font size on Figs 2 and 4, difficulty to distinguish colours and lines on Fig 7j.

We have improved the readability of the figures by increasing the font sizes and enhancing the contrast of lines and colors, particularly in Figs. 2, 4, and 7j. These adjustments make all labels and graphical elements clearer in the revised version.

Thank you for making the code available. I suggest adding a readme to the github directory to help others navigate the code.

We have added a README file to the GitHub repository to help users navigate the code and reproduce the analyses.
